# Capnography as a tool to detect metabolic changes in patients cared for in the emergency setting

**DOI:** 10.1590/1518-8345.1756.2885

**Published:** 2017-05-15

**Authors:** Francisco José Cereceda-Sánchez, Jesús Molina-Mula

**Affiliations:** 1 Doctoral student, Universitat de les Illes Balears, Mallorca, Spain. RN, Servicio de Salud de las Islas Baleares (Ib-Salut), Islas Baleares, Spain.; 2 PhD, Professor, Escuela de Enfermería y Fisioterapia, Universitat de les Illes Balears, Illes Balears, Spain.

**Keywords:** Capnography, Metabolic Diseases, Acidosis, Alkalosis, Carbon Dioxide, Spontaneous Breathing.

## Abstract

**Objective:**

to evaluate the usefulness of capnography for the detection of metabolic changes in spontaneous breathing patients, in the emergency and intensive care settings.

**Methods:**

in-depth and structured bibliographical search in the databases EBSCOhost, Virtual Health Library, PubMed, Cochrane Library, among others, identifying studies that assessed the relationship between capnography values and the variables involved in blood acid-base balance.

**Results:**

19 studies were found, two were reviews and 17 were observational studies. In nine studies, capnography values were correlated with carbon dioxide (CO_2_), eight with bicarbonate (HCO_3_), three with lactate, and four with blood pH.

**Conclusions:**

most studies have found a good correlation between capnography values and blood biomarkers, suggesting the usefulness of this parameter to detect patients at risk of severe metabolic change, in a fast, economical and accurate way.

## Introduction

In the emergency services, to diagnose and evaluate the treatments administered to patients with pathologies as diverse as metabolic or electrolytic changes, hypoxemia and hypercapnia, an arterial blood gas (ABG) test or a venous blood gas (VBG) test is required to assess the oxygenation, ventilation and metabolic status^1^. On the other hand, blood gas analysis is usually not a supplementary test available in outpatient emergency services and in many hospital emergency services, and it is unusual the presence of specific equipments, which requires sending a sample to the laboratory, with a consequent delay in results^2^.

The assessment of blood acid-base balance is performed using aggressive techniques, which require material resources, team time and are not free of potential complications^1^ ^,^ ^3^. Capnography is an alternative method that can help assess the patients' metabolic status in a noninvasive way, in fact, it has been used for years as a quality standard in patient care monitoring in a variety of heathcare areas, including anesthesia and resuscitation, intensive care and emergencies^4^ ^-^ ^6^. Through it, a supplementary monitoring to pulse oximetry is achieved, as capnography provides direct and immediate information on ventilation, whereas pulse oximetry only quantifies oxygenation^7^. With the use of capnography, it is possible to know objectively the patients' metabolic status^1^ ^-^ ^3^, correct installation of orotracheal tube (OTT) in the bronchial tree, quality and effectiveness of cardiopulmonary resuscitation (CPR) maneuvers, restoration of spontaneous circulation during CPR, monitoring of invasive and non-invasive mechanical ventilation and spontaneous ventilation^8^ ^-^ ^10^.

Several revised articles suggest the usefulness of capnography for this purpose, given a good correlation between CO_2_ values at the end of expiration (known as end-tidal CO_2_ or EtCO_2_) and other variables involved in the binomial blood acid-base^1^ ^-^ ^3^ ^,^ ^5^ ^,^ ^10^ ^-^ ^12^. For more than a decade, emergency medical services (EMS) have long been equipped with portable capnographs, according to the last three published editions of the international guidelines on CPR^9^ ^,^ ^13^ ^-^ ^14^, so that capnographs are generally included in the defibrillator-monitors^7^. Therefore, it is interesting to know the potential utility of this parameter to detect these changes, as well as to analyze the variables that potentially influence the EtCO_2_ measurements in spontaneous breathing patients.

**Figure 1:**
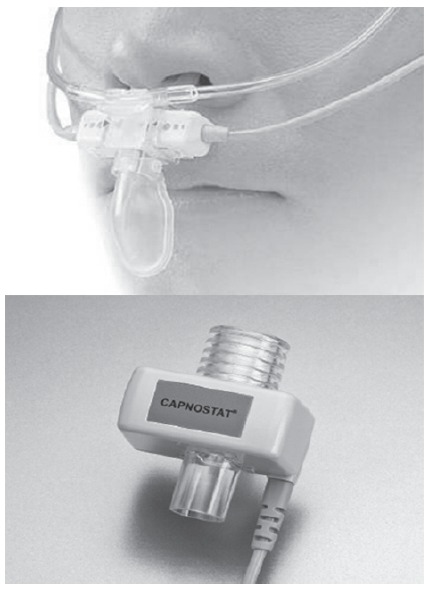
Mainstream adapter for orotracheal tube, on the bottom and on the top, for nasal cannulae with oral portion, aiming the collection of air exhaled through the mouth. As can be seen, the sensor is close to the airway

**Figure 2:**
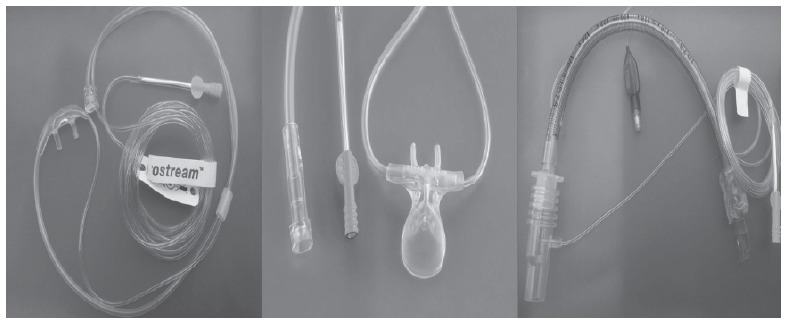
From left to right: simple nasal cannulas, nasal cannulas with oral portion and adapter for OTT in Sidestream systems

The types of capnography infrared sensors used in the current monitors are mainly divided into two types according to their location: mainstream, whose sensor is located near the airway (Figure 1); sidestream, whose sensor is on the monitor, away from the airway, and through a cannula, a small volume of the exhaled air is continuously aspirated (between 100-150ml/min) (Figure 2) and passed into a sensor located on the monitor. In addition, currently, the Microstream technology is also available, a version of the Sidestream that requires even less sample, about 50 ml/min^7^ ^,^ ^15^ ^-^ ^17^. All systems are supplied with adapters for OTT and nasal or oro-nasal cannulas.

Some of the revised studies^1^ ^,^ ^18^ ^-^ ^19^ suggest the usefulness of this parameter for an initial and a rapid screening, both at hospital and outpatient levels, of those patients at high risk of suffering from some severe metabolic change. This indicates the potential of this parameter as a sentinel sign, capable of detecting those patients at highest risk, in order to quickly submit them to the necessary supplementary screening tests and administer an initial treatment as early and specific as possible. Some authors have already defined capnography as the sixth vital sign, with potential to improve risk stratification in the emergency settings^19^. 

The objective of this review is to evaluate the usefulness of capnography for the detection of metabolic changes in spontaneous breathing patients, in the emergency and intensive care settings.

## Methods

An in-depth and structured bibliographical research was carried out from December 2015 to January 2016, in two phases.

Firstly, from the question and objectives of the study, it was obtained the keywords that were translated into documentary language or descriptors on Health Sciences Descriptors (DeCS). The entries *capnography* and *metabolic diseases* were selected as root or primary descriptors, *acidosis* and *alkalosis* as secondary, and their combination with the Boolean operators was set as follows: (capnography[MeSH]) AND (metabolic diseases[MeSH] OR acidosis[MeSH] OR alkalosis[MeSH]).

Considering the areas of knowledge, the following databases were selected for the collection of primary sources: EBSCOhost [which included the databases: MEDLINE Complete, Cumulative Index for Nursing and Allied Health Literature (CINAHL) Complete, Database of Abstracts of Reviews of Effects (DARE), Cochrane Central Register of Controlled Trials (CENTRAL), Cochrane Methodology Register (CMR), NHS Economic Evaluation Database (EED), Health Technology Assessments (HTA), Library Information Science & Technology Abstracts (LISTA), Virtual Health Library (VHL), PubMed, Spanish Medical Index (IME), Spanish Bibliographic Index of Health Sciences (IBECS), Latin American Literature in Health Sciences (LILACS) and Cochrane Library. The selection of articles was limited to all types of publication in English and Spanish over the last 10 years. Those references whose title and content did not meet the inclusion and exclusion criteria were excluded.

 The inclusion criteria were that the article included capnography in the assessment of patients with potential metabolic changes, in its title or abstract; that the article assessed the consistency between the values obtained by capnography and the other parameters included in the blood acid-base balance; and that spontaneous breathing patients were included in the study. The exclusion criteria were that the study did not assess the correlation between the gasometric values of blood acid-base balance and the EtCO_2_ values; that the objective of the study was to assess the correlation between arterial CO_2_ partial pressure (PaCO_2_) and EtCO_2_, only in chronic respiratory patients. In addition, these studies assessed the use of capnography only in patients submitted to invasive mechanical ventilation and only focused on transcutaneous capnography. Table 1 shows the distribution of the articles found, according to the different databases.

**Table 1:** Distribution of articles according to the databases. Palma de Mallorca, IB, Spain, 2016

Database queried	Total of articles found	Articles excluded	Review articles selected
EBSCOhost	19	10	9
Pubmed	10	4	6
IBECS	1	0	1
IME Biomedicine	1	0	1
LILACS	0		
VHL	3	1	2
Total phase 1	22*	12*	10*
Total phase 2 (Guided search)	0	0	9
Total	0	0	19

*Removed repeated articles

In the second phase, a specific research was carried out in order to complete the selection of articles. For this purpuse, some of the citations that the authors of the selected studies used and were relevant for the present study were located and incorporated, as shown in Tables 1 and 2.

**Table 2:** Types of studies included in this review and total articles found for each scoring, according to the Likert scale. Palma de Mallorca, IB, Spain, 2016

Type of study	Total articles	1 Point Likert	2 Points Likert	3 Points Likert	4 Points Likert
Review articles	2				
Retrospective Observational Studies	2				
Prospective Observational Studies	15				
Total according to the Likert Scale		0	1	3	15

The structured search was performed in pairs as well as the final decision to include or exclude a certain study. In preparing of this article, a worksheet was used for the elaboration of a structured summary about each consulted article (introduction, justification, objectives, type of study design, year of completion, sample size, methodology, main results, discussion, limitations, conclusions, observations and recommended bibliography). In this worksheet, the degree of adequacy of each article was assessed using a 4-point Likert scale, according to the criteria and methodological quality of the results presented. The Likert scale scoring was as follows: 1 point if the article was not relevant for the study objectives, 2 points if it was relevant for the justification of theoretical framework of the study, but with poor methodological quality, 3 points if it was relevant for the study methodology, but with uninteresting results for the study, 4 points if it was relevant for the methodology, results, conclusions and theoretical framework.

After completing the two phases of the bibliographical search, the same strategy was repeated by an expert in Documentation Science, using the descriptors and their Boolean combinations over the ten years and the language used in the databases, and the same results were found. Thus, the validity of this review was ensured.

## Results

In the initial phase of this study, 22 articles were selected and 11 critical readings resulted after applying the inclusion and exclusion criteria. These 11 articles were scored according to the Likert scale (Table 2). 

In the second phase of the guided search or snowball sampling, another nine articles were selected, which are presented in Table 2, together with the initial search and scoring obtained according to the Likert scale. Two of the included articles^17^ ^,^ ^20^ had been selected because they analyzed variables such as the correlation between the measuring devices, according to the type of sensor used, Mainstream or Sidestream, which seemed to be important factors in evaluating the possible variables involved in the parametrization of capnography in spontaneous breathing. 

Of the 19 articles finally selected, 17 were from the primary search (89.4%) and two from the secondary search (10.53%). Regarding the distribution according to the study design, 15 were prospective observational studies (88.23%) and two were retrospective observational studies (11.76%), of which five were focused on pediatric patients (29.41%) and the remaining 12 ones were focused on adults (70.59%), as described in Table 3.

**Table 3:** Different variables analyzed in the included articles. Palma de Mallorca, IB, Spain, 2016

Author	n	Cannula used	Type of sensor	Capnograph used	Measurement duration
Pishbin et al.**^(^** **^1^** **^)^**	64	Nc*	Sidestream	Capnocheck**^(r)^** Sleep Capnograp/oximeter.	NK/NA**^†^**
Agus et al.**^(^** **^11^** **^)^**	72	Nc*	Microstream	MDE Escort Prism**^(r)^** monitor	NK/NA**^†^**
Gilhotra & Porter **^(^** **^23^** **^)^**	58	Nc*	Sidestream	Philips M3046A.	1 min
Solana et al.**^(^** **^12^** **^)^**	25	Nco**^‡^**	Microstream	Philips smarth capnoline O2 pediatrics.	30 sec-1min
Nagler et al.**^(^** **^24^** **^)^**	130	Nco**^‡^**	Microstream	Microcap; Oridion.	1-2 min
Kartal et al.**^(^** **^29^** **^)^**	240	O**^§^**	Sidestream	Medlab Cap 10 sidestream.	NK/NA**^†^**
McGillicuddy et al.**^(^** **^30^** **^)^**	97	NK/NA**^†^**	Microstream	Nelcor NBP-70.	NK/NA**^†^**
Soleimanpour et al.**^(^** **^3^** **^)^**	181	NK/NA**^†^**	NK/NA**^†^**	RESPIRONICS device (model number: 7100).	1 min
Yosefy et al.**^(^** **^2^** **^)^**	73	Nc*	Sidestream	OHMEDA Model 4700 Oxycap monitor.	NK/NA**^†^** highest value
Kasuya et al.**^(^** **^17^** **^)^**	60	Nco**^‡^** and Nc*	Mainstream and Microstream	Sidestream (Microcap, Oridion Capnography) Mainstream (cap-ONE; Nihon Kohden)	5 min
García et al.**^(^** **^26^** **^)^**	121	Nco**^‡^**	Sidestream	Pryon SC-300.	cont monit
Cinar et al.**^(^** **^22^** **^)^**	162	adap OTT	Mainstream	EMMA Emergency Capnometer	NK/NA**^†^**
Pekdemir et al. **^(^** **^20^** **^)^**	114	Nco**^‡^** and experimental	Mainstream and Microstream	Nihon Kohden TG-921T3 in Mainstream. Mindray Benewiew T5 monitor, for sidestream	NK/NA**^†^**
Hunter et al.**^(^** **^19^** **^)^**	1088	NK/NA**^†^**	Microstream	LIFEPAK 12 multiparameter defibrillator/monitors.	3-5 ventilations
Hunter et al.**^(^** **^18^** **^)^**	201	Nc* in sidestream, adap OTT Mainstream	Mainstream and Microstream	Capnostream 20 device (Oridion Medical 1987 Ltd).	3-5 ventilations
Delerme et al.**^(^** **^28^** **^)^**	43	Nco**^‡^**	Microstream	Datascope, LaCiotat.	NK/NA**^†^**
Jabre et al.**^(^** **^27^** **^)^**	49	Nco**^‡^**	Microstream	Microcap, Oridion Capnography Inc.	NK/NA**^†^**

* Nc= Nasal cannula; † NK/NA= Did not know/Did not answer; ‡ Nco= Nasal cannula with oral portion; § O= Oral Cannula

The pathologies studied in the pediatric population were: 3 articles on the use of capnography in diabetic ketoacidosis (DKA) and 2 in acute gastroenteritis (AGE). In the adult population, 3 articles were focused on patients cared for in emergency settings due to metabolic changes, 4 on dyspneic patients, 2 on septic/febrile patients, 1 on DKA and finally, 2 assessed the Mainstream and Sidestream systems. The sample sizes of the studies were very variable, ranging from 25 subjects, which was the smallest number of patients included^12^, to 1088 patients, which was the largest sample analyzed^19^. The sample size mean of all studies analyzed was 163.41 individuals, whose distribution can be observed in Table 3.

As for the studies from the secondary search, in one of them^21^, it was carried out a review on the use of capnography protocols in an outpatient service to diagnose septic patients. The other one^15^ is an article on nursing continuing education and updating of knowledge about the different fields of application of capnography.

Regarding the materials used for parameterization of EtCO_2_, it was found four studies that used simple nasal cannulas (Nc); five used nasal cannulas with oral portion (Nco) for the detection of the air exhaled through the mouth; two used adapters of OTT, through which patients with ventilation breathed; three studies did not specify the type of cannula used and; three studies used different cannulae or adapters, according to the type of capnograph used^17^ ^-^ ^18^ ^,^ ^20^.

As for the capnographs used according to their technology, 12 used Sidestream capnographs, out of these, seven were of the Microstream type. Only one study exclusively used the Mainstream^22^ system, another one specified the type of instrument used^3^, but did not prove its technology since the manufacturer's specifications were not revealed, and three used two systems (Mainstream and Microstream) to compare their results^17^ ^-^ ^18^ ^,^ ^20^.

The list of capnographs used is shown in Table 3. In total, 16 capnographs of different brands and models have been used, and the only capnograph used in three studies was the Microcap, Oridion Capnography Inc., Needham, MA. Nine of these articles do not specify the duration of the capnography measurement and eight do. Of these, one performs a continuous monitoring, five perform monitoring for one minute or more, and two measure the EtCO_2_ values after 3-5 ventilations.

Regarding the correlation between the EtCO_2_ values and the values of the variables involved in blood acid-base balance, six of the studies compared these values using venous samples, the remaining 11 studies used arterial blood samples. Nine studies used the Pearson correlation coefficient to analyze the association with PCO_2_, eight with HCO_3_, three with lactate, four with blood pH, one with the *Sequential Organ Failure Assessment* (SOFA). In addition to linear correlation, six studies also analyzed the concordance between the measurements and the results by means of the Bland-Altman Formula (FBA). 

Among the pediatric studies, one of them^23^ found that no patient with EtCO_2_ > 30mmHg had DKA (sensitivity of 1.0, specificity of 0.86), correlation between EtCO_2_ and HCO_3_^¯^ (r=0.72). In this study, these findings can be compared with those of another study^24^, which observed that EtCO_2_> 34mmHg is out of range in relation to values of HCO_3_¯ ≤15mmHg (100% sensitivity), whereas EtCO_2_ ≤ 31mmHg showed 96% specificity in detecting acidosis in pediatric patients with AGE; indicating a significant correlation between EtCO_2_ and HCO_3_ (r=0.80, p<0.0001). 

In their studies^25^, other authors monitored children with AGE and dehydration as well as the evolution of the treatment by capnography when intravenous rehydration was initiated, and they observed an improvement in the correlation between the initial values of EtCO_2_ and HCO_3_^¯^ (r=0.61, p<0.0001), and after treatment initiation (r=0.75, p<0.0001). Another study^26^ also found a good correlation between EtCO_2_ and pH (r=0.88, p<0.0001) and between EtCO_2_ and PCO_2_ (r=0.92, p<0.0001) during continuous monitoring of patients with DKA and, by means of FBA, the limits of agreement between EtCO_2_ and PCO_2_ were defined as 0.8 ± 4.2 mmHg. In a later study^11^, similar results were also obtained with the same type of pediatric patients, between EtCO_2_ and PCO_2_ (r=0.84, p<0.0001), and between EtCO_2_ and HCO_3_^¯^ (r=0.84, p<0.0001). They also assessed the concordance between EtCO_2_ and HCO_3_^¯^ by using FBA and the result was -0.51 ± 2.31 mmHg, and between EtCO_2_ and PCO_2_ was -0.29 ± 4.18 mmHg. 

In studies targeting adults grouped according to the reason of the consultation, the following results were found.

In patients with dyspnea, one study^27^ analyzed the concordance between EtCO_2_ and PaCO_2_, with a mean deviation between the two parameters of 12mmHg. The correlation between the EtCO_2_-PaCO_2_ gradient and the respiratory rate, obtained by FBA, was weak (r=0.21; p<0.014); however, the authors did not analyze the direct correlation between EtCO_2_ and PaCO_2_. On the other hand, in another study^28^, a good correlation was found between EtCO_2_ and PaCO_2_, but the concordance between the two values, by means of FBA, was weak, ranging from -10 mmHg to +26 mmHg. It should be emphasized the results of another previous study^2^, that, on the contrary, found a strong correlation between EtCO_2_ and PaCO_2_ (r=0.792). Similarly, another study^22^ found a strong correlation between EtCO_2_ and PaCO_2_ (r=0.911, p<0.001) and also a good concordance, by means of FBA, 0.5 ± 5 mmHg (95% CI, -1.3165-0.2680). 

Among those studies comparing different capnography systems, it was observed a study^20^ that found: using the Mainstream system, a moderate correlation between EtCO_2_ and PaCO_2_ (r=0.55, p<0.001), by FBA, from -0.6 mmHg to 25.5 mmHg. With the use of the Microstream system, the correlation between EtCO_2_ and PaCO_2_ was r=0.41 (p<0.001), by FBA, ranging from -5.4 mmHg to 24.7 mmHg. In another study^17^ conducted with non-obese postoperative patients, obese with and without obstructive sleep apnea syndrome (OSAS); comparing two measuring instruments and three different cannulae, the following results were obtained in the correlation between EtCO_2_ and PaCO_2_: Non-obese: Mainstream-Nco (r=0.91, p<0.001), Microstream-Nco (r=0.85, p<0.001), Microstream-Nc (r=0.72, p<0.001). Obese patients without OSAS: Mainstream-Nco (r=0.91, p<0.001), Microsteam-Nco (r=0.7, p<0.001), Microstream-Nc (r=0.65, p<0.001). Obese patients with OSAS: Mainstream-Nco (r=0.76, p<0.001), Microstream-Nco (r=0.72, p<0.001), Microstream-Nc (r=0.39, p<0.001). 

Among those studies focused directly on metabolic changes, it was found a study^3^ that is the only one aiming at the detection of DKA in adults. In such a study, it was obtained a moderate correlation between EtCO_2_ and PaCO_2_ (0.572, p>0.0001) and a strong correlation between EtCO_2_ and HCO_3_¯ (r=0.730, p>0.0001), indicating that values of EtCO_2_ > 24.5mmHg were out of the interval for DKA, with sensitivity of 0.90 and specificity of 0.90. On the other hand, in another study^29^ that found a moderate correlation between EtCO_2_ and HCO_3_ (r=0.506), it was obtained values of EtCO_2_ ≤ 25, with a specificity of 84% for acidosis in its sample, and EtCO_2_ ≥37mmHg, with 100% sensitivity for absence of metabolic acidosis. Still, in another previous study^30^, a weak inverse correlation was obtained between EtCO_2_ and SOFA (r= -0.35, p<0.01) and between EtCO_2_ and lactate (r= -0.35, p<0.01) in febrile and potentially septic patients. However, in a later study^18^, a moderate inverse correlation was found between EtCO_2_ and lactate, according to the septic status: in patients with sepsis (r= -0.421, p<0.001), severe sepsis (r= -0.597, p<0.001), and septic shock (r= -0.482, p<0.011). They studied the relationship between EtCO_2_ values and mortality, obtaining in those who died, a mean EtCO_2_ value of 26mmHg (95%CI, 21-30); and for those who survived, a mean EtCO_2_ value of 33mmHg (95%CI, 31-34). In another study of the same authors mentioned above^19^, aiming at the detection of septic patients, it was observed a moderate correlation between EtCO_2_ and HCO_3_¯ (r=0.429; p<0.001), as well as between EtCO_2_ and lactate (r= -0.376, p<0.001), which was only detected in 89 patients (n=201). In this case, the mean EtCO_2_ value in patients who survived was 34mmHg and the mean EtCO_2_ value in those who died was 25mmHg. Finally, there are the results of the last published article^1^, which found a strong correlation between EtCO_2_ and HCO_3_¯ (r=0.869, p<0.06), a weak correlation between EtCO_2_ and pH (r=0.795, p<0.001), and a weak correlation between EtCO_2_ and base excess by ABG analysis (r=0.346; p<0.006).

## Discussion

Most of the studies show that capnography has proven to be a gold standard in the urgency and emergency settings, and its complementarity is evidenced along with pulse oximetry, in the monitoring of patients' breathing, circulation and metabolism. In individuals with normal lung function, a 2-5 mmHg gradient difference between EtCO_2_ and PaCO_2_ is accepted regardless of age^16^ ^,^ ^31^ ^-^ ^32^. The vast majority of the analyzed studies from the primary search showed a high correlation between the capnography values and the blood values of PCO_2_ and/or HCO_3_^¯^. 

It is noteworthy that all studies on the pediatric population, both aimed at the detection of DKA^11^ ^,^ ^23^ ^,^ ^26^ and those performed in patients with AGE^24^ ^-^ ^25^, show a strong correlation between the EtCO_2_ values and PCO_2_ or HCO_3_^¯^. These studies have all been performed on venous samples, which is consistent because they have been carried out on child population and are less invasive tests. 

These results are considered as paradoxical because the differential physiological gradient between EtCO_2_ and venous CO_2_ pressure (PvCO_2_), should be greater than the gradient between EtCO_2_ and PaCO_2_, as the mean difference between PaCO_2_ and PvCO_2_ is 6-8 mmHg (40mmHg PaCO_2_ vs 48mmHg PvCO_2_)^15^ ^-^ ^16^. According to these studies^11^ ^,^ ^24^, EtCO_2_ is a valid and reliable system for use in the pediatric population, and may even help to reduce costs, as it diminishes the blood tests, emphasizing that it is not possible to completely abolish the latter as reliable tests for confirmation of the results. 

On the other hand, in the adult population, no correlation and/or concordance was found between the study variables, discouraging the use of this system to assess the patient's metabolic and/or ventilatory status, according to several studies^20^ ^,^ ^27^ ^-^ ^28^ ^,^ ^30^. The final diagnoses of the patients included in these studies^27^ ^-^ ^28^ were associated with chronic respiratory or cardiac diseases, which directly influences the physiology and the EtCO_2_ values. However, a good correlation was found in previous studies^2^ ^,^ ^22^ with similar distribution of pathologies. 

Special attention should be given to the results of one of the studies^20^, in which patients with dyspnea treated in an emergency service were evaluated, including a group of individuals without this respiratory disease in the sample, in order to reduce the bias, according to the variables and measuring instruments. These authors found a moderate correlation between EtCO_2_ and PaCO_2_, despite of using a Microstream and Mainstream system, indicating a good correlation between the measurements with the use of a Mainstream system, which may also be related to the results of a previous study^22^ that used a Mainstream system. 

Having the sensor near the airway seems to reduce the chance of mixing atmospheric air, just as Sidestream systems tend to increase dead space through the aspiration tube. In fact, in the conclusions of a study^20^, it is suggested that the low correlation found in the results is due to the measuring systems and methods. In this sense, it can be noticed that in the results of another study^17^, which assessed three types of patients with different pathologies, all measurements were performed using a Mainstream and Microstream system, along with different cannula models, and a strong correlation was found. This was the strongest correlation found with the use of a Mainstream system, which presented a good correlation in the non-obese patients without OSAS since they had a better pulmonary function.

On the other hand, based on another study^30^ with potentially septic patients, it is not possible to draw the same conclusions, since the final diagnostic of patients was not revealed, although patients with chronic respiratory disease were excluded. This study did not recommend the use of capnography as a tool for decision making, but it mentions the feasibility of its use for monitoring in the emergency services. This study also did not reveal the type of cannula used or the duration of EtCO_2_ measurement, which makes its reproducibility difficult and may lead to measurement imprecisions.

Studies focused purely on the detection of metabolic changes in adults^3^ ^,^ ^18^ ^,^ ^30^, aim to ratify its practical utility as a tool for clinical decision making. A study^3^ seems to show its real potential use as a predictive tool in emergency services as well as an indicator of acidosis or not. In another study^18^ on patients with suspicion of sepsis, a better correlation with lactate levels was observed than in the previous study^30^, which may be because the sample in the last study^18^ was twice as large (n=97 vs n=201). In addition, patients who needed OTT were included and two different measuring systems were used; a Mainstream system for patients with OTT, and a Microstream system for those with spontaneous breathing. Checking the type of capnography, it was verified the use of the Microstream system, considering that the capnograph does not have the two systems (Mainstream and Microstream). It was noticed a transcription error and, in fact, a Microstream adapter for OTT was used. These authors^18^ also assessed the correlation of EtCO_2_ values with mortality and lactate, indicating their use to predict mortality and the presence of septic status in these patients. 

In a later study^19^ ^),^ with a much extensive sample (n=1088), the same authors also found a strong correlation. In addition, they analyzed and compared the values of normal vital signs in relation to EtCO_2_, the latter parameter being the most predictive and consistent value to indicate mortality in the outpatient environment, hence they designated it as the sixth vital sign. In this later work^19^, the authors indicate the need for advanced life support maneuvers and the use of Nc and OTT as inclusion criteria. It is understood that most of the data collected shall refer to patients with spontaneous ventilation, since the authors compared the predictive values of all vital signs with those values measured by capnography.

In future studies, correlations between the different groups of patients should be better analyzed according to the alkalosis or acidosis status of metabolic or respiratory origin, since strong correlations have been found between EtCO_2_ and HCO_3_¯ and between EtCO_2_ and PaCO_2_, especially in alkalotic patients. This is an important factor to be taken into account, since the physiological compensatory response of metabolic acidosis is respiratory alkalosis, which can also occur in other common pathologies such as anxiety crisis.

In addition, it should be noted that EtCO_2_ values are influenced by various physiological factors, such as tissue metabolism, venous circulation, cardiac output, alveolar perfusion, and alveolar ventilation per minute^22^. Any change of any of these factors will directly affect the EtCO_2_ values, so there is the feasibility of getting low values, which are indicative of acidosis, whereas the problem may be changes in the perfusion or ventilation rather than metabolism. 

According to the literature reviewed, it should also taken into account that in the case of a patient with extremely low EtCO_2_ values and severe symptomatology, it can be assumed that he probably suffers from some severe pathology, which is causing this alteration and will require emergency medical assistance. This may also be the case of a patient with pulmonary thromboembolism, severe or respiratory heart failure, hemorrhagic shock, etc. For this reason, in several studies (especially pediatric), patients with cardiac, pulmonary or renal problems were excluded.

In general, it was observed a deficit in the control group with healthy patients in all the studies assessed, lack of information on the measuring instruments and duration of the measurements, for example, eight studies did not specify the duration of the measurements. The sample sizes were small and convenience sampling was used, as most authors indicated in their limitations. It is not possible to extrapolate the results in relation to the measuring equipment because several capnograph models were used (Table 3). All these factors, added to the variability of the measurements, types of patients and pathologies, characterize a heterogeneity that does not allow making viable comparisons between the studies. However, among all the studies, only four ventured to adjust the cut-off values^3^ ^,^ ^23^ ^-^ ^24^ ^,^ ^29^ ^)^ by means of a Receiver Operating Characteristic curve (ROC), finding average values, from 24.5 mmHg, the lowest, to 36 mmHg, the highest, as the upper cutoff point to rule out the possibility of acidosis. Values from 24.5 to 31 mmHg could indicate the probability of acidotic status. As can be seen, the amplitude of the upper cutoff values was 11.5 mmHg, whereas the lower cutoff values show an amplitude less than 6.5 mmHg between the values found. Due to the high variability in the data available up to now, it is not possible to recommend precise cutoff values for the use of this parameter in clinical decision making.

## Conclusion

Most of the studies found results with a good correlation between EtCO_2_ and HCO_3_^¯^, or between EtCO_2_ and blood PCO_2_. Although further studies are needed to evaluate these associations, it is possible to suggest that the scientific evidence supports the potential use of capnography as a new sign, biomarker or complementary sentinel parameter to detect those patients with severe disease and it can be easily implemented for use in spontaneous breathing patients. 

While EtCO_2_ values above 24.5-36 mmHg appear to exclude metabolic acidosis status, EtCO_2_ values less than 24.5-31 mmHg are indicative of acidotic status. Therefore, low capnography values, especially less than 24.5 mmHg in patients with other signs or symptoms associated with some severe pathology, may indicate the need for more specific tests and avoid delays in assistance, thus reducing morbidity and mortality in the emergency settings.
